# The Impact of Specific Psychological Characteristics on Decision-Making Under the Different Conditions of Risk Self-Assessment

**DOI:** 10.3389/fpsyg.2022.779246

**Published:** 2022-03-15

**Authors:** Zhijuan Liang, Ximing Liao, Huajian Cai

**Affiliations:** ^1^Institute of Psychology, Chinese Academy of Sciences, Beijing, China; ^2^Department of Psychology, University of Chinese Academy of Sciences, Beijing, China; ^3^Department of Human Resource, China Mobile Communications Group Co., Ltd., Guangzhou, China

**Keywords:** risk assessment, decision-making, curiosity, optimism, courage

## Introduction

In the face of challenging and novel activities or events (e.g., bungee jumping, academic competitions, new project developments), people can choose to participate or not. Decision-making behavior is the result of multiple factors, and assessment of risk activity is one such factor in behavioral decision-making (Zuckerman, 2007). Activity characteristics, personal characteristics, and social situation factors can also influence decision-making. The key determinants of decision-making include cognitive and affective states (Yuen and Lee, 2003), motivation (Ramey et al., 2016), and personality traits (Skeel et al., 2007). Personality traits can include sensation seeking (Zuckerman, 2007), openness to experience (Peterson and Seligman, 2004), and impulsiveness (Lejuez et al., 2002).

Individual differences can be expected to drive effects on decision-making processes and outcomes as well as the interaction between individual differences and other factors (Appelt et al., 2011). Curiosity, optimism, and courage are all strongly associated with uncertainty or risk (Loewenstein, 1994; Lopez, 2007; Carver and Scheier, 2014). Therefore, in this article, we focus on these three psychological characteristics and risk assessment in decision-making. We propose that there are important psychological characteristics that drive people to make decisions and behave under different conditions of risk self-assessment.

## Risk Assessment

The decision to participate in an activity depends on its benefits, expected positive outcomes, and risks. Risk is inherent in every decision that we make and is defined as the possibility of facing undesirable consequences as a result of an activity or situation and the probability of suffering harm or loss (Vlek and Stallen, 1981; Vlek, 2004; Zuckerman, 2007). Yates and Stone (1992) identified six categories of loss: financial loss (money), performance loss (for a product), physical loss (from temporary discomfort to death), psychological loss (self-esteem), social loss (esteem of others), and time loss.

Since risk is a multifaceted concept, risk assessment requires complex methodologies (Wilson and Crouch, 1987). Individual risk assessment is a subjective matter that directly affects decision-making related to risky behavior and may differ from objective risk indicators (Zuckerman, 2007). The two main systems currently used to understand and assess risk are the “analytic system,” which brings logical, rational, and scientific considerations to risk management, and the “experiential system,” which represents risk as a feeling, an individual's quick, instinctive, and intuitive response to danger (Slovic et al., 2004, 2005). In practice, the ultimate decision is usually based on a limited perception rather than on an understanding of the full value and meaning of the risk.

The result of risk assessment is the synthesis of risk propensity and risk perception. Risk propensity is the “willingness to take risks” (MacCrimmon and Wehrung, 1990) and is considered to be a general attitude toward any type of risk. Its impact is thus horizontal in all areas. Risk perception is understood to be an individual's assessment of the degree of risk in a situation (Baird and Thomas, 1985) and may influence risk behavior to varying degrees, depending on the domain (de-Juan-Ripoll et al., 2021). Both risk propensity and risk perception have been shown to exert a strong influence on risk decision behavior (Sitkin and Weingart, 1995). A study conducted by Keage and Loetscher (2018) found that different aspects of the individual—cognitive, emotional, social, and cultural factors, experiences of previous activity, knowledge of others' past experiences, and expertise in the subject—are likely to influence risk perception.

In psychology, riskiness usually denotes a directional effect (i.e., a high chance of loss), whereas uncertainty does not show a similar directional connotation (De Groot and Thurik, 2018). Reference physical activities are classified as low, medium, and high risk according to their degree of harm to the body (Zuckerman, 1983; Gomà-i-Freixanet, 2004). In this article, we assumed that individual risk self-assessment results included low, medium, and high risks according to the degree to which an activity impacts individual negative outcomes (including physical and psychological consequences). Low risk means that the probability of fatal injury or significant loss is very low. Medium risk means that the probability of injury is higher than the risk of death or the uncertainty of loss. High risk means that the possibility of serious injury or death and other significant loss is very high, usually accompanied by the fear around failure (Gal and Rucker, 2020). There is no directional meaning to the uncertainty here, including the uncertainty of results and information (Pathak et al., 2021). Behavioral economists call such uncertain decisions “decisions under ambiguity” in which outcomes are not described by a probability distribution (Brand et al., 2007). Although there are frequent risks in daily activities, most people do not sense the risk in most activities and consider those activities to be low risk.

## Psychological Characteristics

### Curiosity

Curiosity is considered a positive emotional-motivational system that pursues novelty and challenge and is a characteristic of personal growth and psychological strength (Kashdan et al., 2004; Peterson and Seligman, 2004; Gallagher and Lopez, 2007). Curiosity arises from the stimulating properties of novelty, complexity, uncertainty, and conflict (Berlyne, 1978). Curiosity can also be interpreted as a cognitively induced deprivation resulting from (a) the perception of gaps in knowledge or understanding, (b) a desire for new information, knowledge, and experience, and (c) sensory stimuli that stimulate exploratory behavior to resolve uncertainty or experience the unknown (Loewenstein, 1994; Grossnickle, 2016; Litman, 2019). Different types of curiosity include diverse and specific curiosity (Berlyne, 1960) and state and trait curiosity (Silvia, 2008a).

In the widely used five-dimensional curiosity scale (Kashdan et al., 2020), thrill-seeking is a dimension of curiosity with risk-taking manifestations. However, a highly curious individual does not necessarily enjoy and seek out new environments with high physical risk or intellectual stimulation; in fact, highly curious people are more likely to recognize, pursue, and immerse themselves in novel and challenging experiences (Kashdan et al., 2004). Stress tolerance, which is one dimension of curiosity, is the tendency to deal with anxiety in the face of new events. Some people not only endure stress but are willing to accept social, physical, financial, and legal risks to gain new experiences. A person's curiosity and exploratory behavior depend, in part, on expectations of outcomes, such as risk assessment and depth of knowledge (Peterson and Seligman, 2004).

Specific aspects of the environment (e.g., perceived threat, autonomy support) and activities (e.g., competition, meaning) influence state curiosity. Mysterious, novel, complex, uncertain, and/or ambiguous events often arouse interest and curiosity (Berlyne, 1962; Silvia, 2008b). To satisfy curiosity, humans may be inclined to perform additional actions beyond the requirements of the task. For example, when the risk is manageable or safe, curiosity drives people to look at pictures that elicit disgust (Hsee and Ruan, 2016).

### Optimism

Optimism has been defined as the cognitive disposition to expect favorable outcomes (Scheier and Carver, 1985) and has motivational implications (Carver and Scheier, 2014). In high-risk environments, optimists respond to losses with a greater willingness to participate and a tendency to mark losses as close to victory, whereas pessimists are more likely to generate appropriate negative risk expectations and leave risky environments (Gibson and Sanbonmatsu, 2004).

Risky behavior can cause stress, and optimism has been shown to reduce the impact of stressors on mental functioning. There is a positive link between optimism and a broadly participatory and problem-focused response (Nes and Segerstrom, 2006). Optimism predicts more problem-centered coping with controllable stressors and more emotion-centered coping with uncontrollable stressors. Optimism predicts positive attempts to change and adapt to a stressful environment, reflecting flexible engagement (Carver et al., 2010). Optimists are less likely to use avoidance strategies such as denial and abandonment (Segerstrom et al., 1998; Carver and Scheier, 2014).

### Courage

Peterson and Seligman (2004) identified courage as one of the six core human virtues, the other five being justice, humanity, temperance, wisdom, and transcendence. Courage includes bravery, persistence, integrity, and vitality and is the virtue of an individual who is willing to face danger and work hard for a goal. Woodard (2004) proposed that courage is the ability of an individual to act out of good intentions, and courage has also been known to ward off fear. Shelp (1984) describes courage in the following way:

The disposition to voluntarily act, perhaps fearfully, in a dangerous circumstance, where the relevant risks are reasonably appraised, to obtain or preserve some perceived good for one's self or others recognizing that the desired perceived good may not be realized (p. 354).

The three characteristics of courageous action are as follows: being guided by correct values, voluntary risk-taking, and realistic risk-taking (Lopez, 2007). Researchers have concluded that courage in an organizational context has five characteristics. These are the freedom to choose, experience risk, assess risk, pursue high goals, and be aware of fear (Kilmann et al., 2010). Through qualitative research, Rate et al. (2007) identified four dimensions of courage, intentionality/deliberation, the presence of personal fear, noble/good acts, and known substantial personal risk. Other studies have also highlighted the link between courage, fear, and risk (Woodard, 2004; Goud, 2005; Rate et al., 2007; Norton and Weiss, 2009). Courage may be expressed in the situation as (a) not feeling fear at all, (b) feeling fear but overcoming it to take action, and (c) acting with fear but disregarding it.

Putman (1997) divided courage into three types—physical courage, moral courage, and psychological courage. Physical courage is a type of courage that overcomes the fear of bodily harm or death to save others or oneself. Moral courage means maintaining moral integrity or authenticity at the risk of losing a friend, job, privacy, or reputation. Psychological courage includes the kind of courage required in the face of debilitating diseases or destructive habits or circumstances. In the process of being courageous, the situation may be so urgent that it is fraught with danger and uncertainty but requires a quick reaction (Osswald et al., 2012). The process of evaluating the courage of others involves assessing elements of courage, such as individual fear, the degree of risk, subjective intention, and so on (Rate and Sternberg, 2007).

## Risk Assessment and Psychological Characteristics

We present the idea that different degrees of risk assessment link with psychological characteristics to influence behavior. It was suggested that different psychological characteristics play various roles under different risk degrees of self-assessment. Specifically, curiosity potentially drives behavior under the condition of low self-rated risk; optimism can drive the participation of the activity under the condition of medium self-rated risk; courage may act as a substantial force to goad participation under the condition of high self-rated risk.

When the risk is self-assessed as low level, it is generally considered a safe state whereby curiosity leads to exploration and experimentation. According to Spielberger and Starr's optimal stimulus/two-process theory, when curiosity is stronger than anxiety, individuals tend to explore their environment (Peterson and Seligman, 2004). Being an intrinsic motivation for information processing, curiosity is determined by the learning progress or information that the cognitive system expects to obtain (Van de Cruys et al., 2021). Information seeking behavior is driven and motivated by individual awareness of the difference between current and target uncertain states (Gottlieb et al., 2013; Van de Cruys et al., 2021). Previous studies have shown that curiosity levels may be affected or suppressed when individuals are uncomfortable with uncertainty or believe that they have a high probability of failure (Hulme et al., 2013; Jirout, 2020). Therefore, exploratory behavior that stems from the expectation of acquiring information and interest in new experiences can be better realized in low-risk contexts.

When risk self-assessment is uncertain, optimists expect things to go their way, believing that good things will occur as opposed to negative events (Scheier and Carver, 1985). Therefore, in the face of the variability and unpredictability of future events, optimism makes it easier to approach action by predicting the future. Optimism induces confidence in people to achieve goals. In addition, more control and persistence can be achieved through the behavioral self-regulation model (Scheier and Carver, 1988). Goal-directed action is guided by a series of negative feedback loops. When obstacles arise in the desired goal path, the individual assesses the likelihood of overcoming the obstacles. Optimistic individuals believe that the ideal outcome is attainable; therefore, they often face adversity while maintaining a positive attitude, thereby increasing the persistence of goals and the realization of goals. Optimism tends to approach goal-related tasks through expectations for the future and confidence that goals can be attained. Specifically, optimists tend to be problem-centered in dealing with controllable stressors and emotion-centered in dealing with uncontrollable stressors (Carver et al., 2010).

When the self-rated risk is high, or even when danger is certain, it is often believed that curiosity, optimism, and courage may work concurrently. However, the main driving force is courage. Courage can help people overcome fears and take risks, even when they are scared or anxious, when self-assessed risks and actual risks are high, when the outcome is uncertain, and when the certainty of a bad outcome or the likelihood of failure is high. Individuals trigger psychological and/or physiological fear responses based on their perception of risk, and courage promotes brave behavior to achieve a specified purpose by facing fear and trying to reduce the level of fear and positive thinking (Hannah et al., 2007). It is a cognitive, voluntary psychological process of implementing change to achieve positive outcomes. Courage involves the desire for positive outcomes, which may be a betterment of one's environment, cognitions, or behavior (Gruber, 2011). This leads to a predictable sense of confidence about acting in the future with the strength of character previously demonstrated in the face of fear, so an individual will thus be willing to take more risks.

These three psychological characteristics are types of approach motivation that are associated with risk and encompass a degree of expectation (Carver et al., 2010; Shaw et al., 2011; Van de Cruys et al., 2021). A major difficulty in the self-assessment of risk is the quantification of subjective uncertainty, which may be caused by insufficient and inaccurate information, inadequate risk assessment ability, and so on. Self-reported measures of uncertainty based on objective stimuli may contain inconsistencies with actual objective conditions, ranging from severe misestimates to approximations. Actions driven by curiosity, optimism, and courage after accumulation are expected to improve risk self-assessment ability over time.

## Discussion

We propose that the role of these psychological characteristics in risk-oriented decision-making may explain the psychological factors that drive people to act in different contexts. This perspective will provide a novel approach to understanding individual differences related to behavioral decision-making, identifying factors that potentially influence decision-making, and enhancing the overall understanding of risk-taking. After being validated by sophisticated empirical studies, the theory is expected to be applied to organizational management, education and development, and other fields. Determining the psychological characteristics needed for risk decision-making can provoke decision-makers to consider their own advantages and disadvantages in the specific decision-making process according to their psychological characteristics and boost their decision-making ability. Studies have shown that the desire for courage motivates individuals to make riskier choices in important life decisions (Gal and Rucker, 2020).

A variety of scenarios can be used to test this idea. When playing in the amusement park, many people will choose to go on the roller coaster. Person A, with their fear of heights, may feel that the game is very risky but possesses enough courage to ride the roller coaster. Person B may think that the game was new but not dangerous, so they ride the roller coaster out of curiosity. Their decision-making and behaviors are similar in that both person A and person B ride the roller coaster, but two different psychological resources provide the main driving force based on the different risk self-assessments—courage on the part of person A and curiosity on the part of person B. From the perspective of the above examples, it is possible to gain a better understanding of the drivers of participation in an activity.

This overview is not an integration of related resources but a list of possible drivers of decision-making under the conditions of risk self-assessment. This article also presents possible directions for designing and evaluating intervention strategies (e.g., fostering curiosity, optimism, and courage to facilitate participation in activities) concerning self-rated risk levels and psychological characteristics. Other psychological characteristics, such as grit (Duckworth et al., 2007) and self-efficacy (Bandura, 1982), may be investigated in future research. Boundary conditions can be clarified to explore the relationship between different psychological characteristics and behavior decisions. Other topics for future research include assessing how curiosity, optimism, and courage interact with risk perception and how different psychological characteristics interact. The role of different psychological characteristics in triumphing over negative emotions such as fear and anger, which are often triggered by uncertainty and risk, is also a relevant research subject.

## Conclusion

Curiosity, optimism, and courage may be the major drivers of behavioral decision-making under the conditions of different self-evaluating risk levels. There are, of course, other psychological characteristics that influence decision-making, and we are open to examining them to contribute to existing research on the topic.

## Author Contributions

ZL was the primary individual on manuscript composition. XL contributed to the writing of the manuscript. HC contributed to the supportive materials. All authors contributed to the article and approved the submitted version.

## Conflict of Interest

XL is employed by China Mobile Communications Group Co., Ltd. The remaining authors declare that the research was conducted in the absence of any commercial or financial relationships that could be construed as a potential conflict of interest.

## Publisher's Note

All claims expressed in this article are solely those of the authors and do not necessarily represent those of their affiliated organizations, or those of the publisher, the editors and the reviewers. Any product that may be evaluated in this article, or claim that may be made by its manufacturer, is not guaranteed or endorsed by the publisher.
